# Comparison of clinical parameters, microbiological effects and calprotectin counts in gingival crevicular fluid between Er:YAG laser and conventional periodontal therapies: A split-mouth, single-blinded, randomized controlled trial

**DOI:** 10.1097/MD.0000000000009367

**Published:** 2017-12-22

**Authors:** Yue Wang, Weiwei Li, Li Shi, Fengqiu Zhang, Sun Zheng

**Affiliations:** a Department of Periodontology; b Department of Oral Medicine, Beijing Stomatological Hospital, Capital Medical University; c Department of Stomatology, Beijing Chongwen Hospital of Stomatology, Beijing, China.

**Keywords:** calcium-binding proteins, chronic periodontitis, lasers, microbiology, nonsurgical periodontal debridement

## Abstract

**Background:**

The erbium-doped yttrium, aluminum, and garnet (Er:YAG) laser is thought to be the most promising laser for periodontal treatment; however, its application is still under consideration. The aim of this study was to compare Er:YAG laser monotherapy with conventional scaling and root planing (SRP) for chronic periodontitis using clinical parameters, the detection rate of periodontal pathogens, and the calprotectin level in gingival crevicular fluid.

**Methods:**

Twenty-seven participants with moderate-to-advanced chronic periodontitis were included. In a split-mouth design, the 2 half-mouths of each participant were randomly assigned to Er:YAG laser or SRP (combination of ultrasonic and manual instruments) treatment. Clinical parameters were recorded at baseline, 6 weeks, and 3 and 6 months after treatment. At the same time points, gingival crevicular fluid was collected to analyze the detection rate of 6 periodontal pathogens by polymerase chain reaction and the levels of calprotectin by enzyme-linked immunosorbent assay.

**Results:**

Both treatment groups showed significant reductions in probing depth (PD), bleeding index (BI), and clinical attachment level (CAL) from baseline to 6 months. For sites with 4 mm ≤ PD ≤ 6 mm at baseline, SRP resulted in a greater reduction in PD and CAL than Er:YAG laser treatment, and the difference remained at 6 months post-treatment (*P* = .01 and *P* < .01, respectively). For sites with PD ≥7 mm at baseline, the clinical parameters showed similar results between the 2 groups. SRP resulted in a lower detection rate of *Porphyromonas gingivalis* at 6 months post-treatment. The levels of calprotectin were significantly decreased from baseline to 6 months in both groups, without a significant difference between the groups.

**Conclusion:**

For mild pockets, conventional SRP may still be the preferred choice. For deep pockets, Er:YAG laser treatment could be an effective alternative. Studies are needed to explore more advanced instruments and new application methods for the Er:YAG laser for periodontal treatment in deep pockets.

## Abstract 2

Supplemental Digital Content is available in the text

## 1 Introduction

Chronic periodontitis is a common inflammatory disease that can result in gingival inflammation, alveolar bone absorption, tooth mobility, and tooth loss. Chronic periodontitis is mainly caused by subgingival microbiota infections combined with the host inflammatory response.^[1]^ The primary goal of chronic periodontitis treatment is to remove the bacterial biofilm and calculus from the teeth and obtain good root surface biocompatibility.^[2]^ For many years, scaling and root planing (SRP), using a combination of hand curettes and ultrasonic scalers, has become the standard treatment for periodontal disease, resulting in beneficial outcomes.^[3]^ However, such conventional treatments have limitations, such as difficulty accessing deep periodontal pockets and other anatomical structures.^[4,5]^

Light from an erbium-doped yttrium, aluminum, and garnet (Er:YAG) laser, which has a wavelength of 2940 nm, is highly absorbed by water and hydroxyapatite to form a micro-explosion, performing the clearance of soft and hard tissues without causing heat damage.^[6]^ It has been reported that an Er:YAG laser can effectively remove calculus and diseased cementum without thermal damage^[7,8]^ and with good bactericidal effects.^[9]^ Several studies and 2 systematic meta-analyses have indicated similar outcomes in terms of clinical parameters between Er:YAG laser treatment and SRP in the short term.^[10–17]^ However, most studies were based on comparisons between Er:YAG laser monotherapy and either manual or ultrasonic SRP alone. The number of comparisons between Er:YAG laser treatment and the combination of ultrasonic and manual instruments, which is the most common nonsurgical treatment, is still limited, and the results are controversial.^[10,18,19]^ Thus, additional evidence is needed to comprehensively evaluate the effects of Er:YAG laser treatment in comparison with the commonly used SRP treatment.

Therefore, the objective of this randomized, split-mouth controlled clinical study was to compare the effects of Er:YAG laser monotherapy and common SRP therapy (combination of ultrasonic and manual instruments) for chronic periodontitis over a 6-month follow-up period with regard to 3 aspects: clinical parameters, the detection rate of periodontal pathogens, and the calprotectin level in gingival crevicular fluid (GCF) as an immune-response inflammatory biomarker.

## 2 Methods

### 2.1 Study design

This study was a randomized, single-blinded, split-mouth, controlled prospective clinical trial. In a split-mouth design, 2 half-mouths of each participant were randomly assigned to the Er:YAG laser monotherapy or SRP; thus, 1 half-mouth of each participant was paired with the other half-mouth from the same participant and served as its control.

### 2.2 Participants

All participants were recruited from the Department of Periodontology, Beijing Stomatological Hospital, Capital Medical University, between September 2013 and October 2016. The Ethics Committee of Beijing Stomatological Hospital evaluated and approved the study protocol (Protocol: 2013–02). Before the study, all participants provided written informed consent.

Inclusion criteria were age ≥18 years; teeth number ≥16; a diagnosis of generalized chronic periodontitis based on the classification of the World Workshop 1999,^[20]^ probing depth (PD) ≥4 mm and clinical attachment level (CAL) ≥2 mm for at least one-third of all mouth sites, and a full-mouth intraoral radiograph was taken to be sure that at least one-third of all approximal sites had bone loss; good general health. Exclusion criteria were periodontal therapy within the previous 6 months; taking antibiotics, steroids, or anti-inflammatory agents within the previous 3 months; current or previous smokers; pregnancy; and systemic diseases, such as diabetes, cardiovascular diseases, or blood diseases.

The sample size was calculated considering 90% power, 5% level of significance, 0.5 mm significant difference, and 0.65 mm standard deviation (SD) for PD, using the PASS 11.0 software package for paired means power analysis. At least 20 patients were required for the study. Additional patients were enrolled in the study to compensate for loss during follow-up.

### 2.3 Oral hygiene program

One week before treatment, all participants were given oral hygiene instructions that reinforced the use of a soft manual toothbrush, dental floss, and interproximal brushes based on individual needs, and also a professional full-mouth supragingival debridement including ultrasonic cleaning and polishing by a single operator, who was blinded to the allocation. The same procedure was performed during recall visits at 6 weeks, and 3 and 6 months after treatment.

### 2.4 Treatment

#### 2.4.1 Er:YAG laser group

An Er:YAG laser (LAEDL001.1, Doctor Smile, Italy) was applied at a pulse energy of 160 mJ and a frequency of 10 Hz with water irrigation. The laser beam was delivered by the hand piece configured with the laser equipment, and the fiber tip was chisel-shaped (1.1 mm × 0.5 mm). During treatment, the fiber tip was held at an angle of 15° to 20° to the root surface and was moved in a coronal to apical direction, with overlapping parallel paths.

#### 2.4.2 Conventional SRP group

The ultrasonic treatment was performed with an ultrasonic hand piece (P5, Satelec, France) and metal tip (H3, H4) at medium or low power, and the manufacturer's instructions were strictly followed. Gracey curettes (Gracey, SG # 5/6, 7/8, 11/12, 13/14, Hu-Friedy) were then used for root surface planing.

All sites with an initial PD ≥4 mm received treatment. The 2 different treatments for each patient were performed at 2 times, and the interval was 1 week. For both groups, the endpoint of the treatment occurred when smooth and thoroughly debrided root surfaces were detected by a pointed probe. Both groups’ treatments were performed by a single experienced operator. To prevent operator bias, another experienced periodontist verified the clinical endpoint. There was no time limit for treatment.

### 2.5 Clinical measurements

Clinical measurements were performed before and at 6 weeks, 3 months, and 6 months after treatment. The plaque index (PLI, Turesky–Gilmore–Glickman modification of the Quigley–Hein index),^[21]^ bleeding index (BI, Mazza index),^[22]^ PD, and CAL (distance from the cemento-enamel junction to the bottom of the pocket) were assessed at 6 sites per tooth.

All clinical examinations were performed by the same examiner. Intraexaminer reliability was determined by 2 measurements performed on 10 patients 5 to 7 days apart. The intraclass correlation coefficient value for PD and CAL were 0.97 and 0.91, respectively.

### 2.6 GCF sampling and processing

Sites with BI ≥2 and PD ≥5 mm at baseline were sampled before and at 6 weeks, and 3 and 6 months after treatment. Before sampling, supragingival plaque was gently removed, and the tooth surface was isolated by cotton rolls and gently dried with an air gun. Absorbent paper points (40#, Meita, South Korea) were carefully inserted into the sampling sites until slight resistance was felt and were held there for 30 seconds. Paper points contaminated by blood or saliva were discarded. The volume of GCF was calculated as the weight difference before and after sampling at a ratio of 1 g/mL. Sampled paper points were then placed separately in sterile tubes and stored at −70°C for further analysis.

Before analysis, the samples were thawed at room temperature, and phosphate buffered solution (pH 7.4, L) was added to achieve a 100-fold dilution. After thorough mixing and centrifugation, the sediment was collected to analyze the microorganism content, and the supernatant was collected to analyze the calprotectin level. All the GCF sampling was performed by the same examiner as performed by the clinical examination, and all the detection was performed by another examiner.

### 2.7 Detection of microorganisms

DNA was extracted from the GCF using a genomic DNA extraction kit (DP302, Tiangen, China) according to the manufacturer's instructions. The samples were analyzed to detect *Porphyromonas gingivalis* (Pg), *Tannerella forsythia*, *Treponema denticola*, *Prevotella intermedia*, *Prevotella nigrescens*, and *Fusobacterium nucleatum* using standard PCR methods.^[23,24]^ The primers for the 6 microorganisms were based on the 16S rRNA gene (see Table, Supplemental Content, which presents the primer sequences for 6 periodontal pathogens).

### 2.8 Detection of calprotectin

Calprotectin levels were assayed using commercially available ELISA kits (CSB-E12149h; Cusabio, China) and strictly following the manufacturer's instructions.

### 2.9 Statistical analysis

The SPSS 19.0 software package was used for the statistical analysis. Qualitative data are reported as frequencies or percentages, and quantitative data are reported as the means and SD. Normality was assessed with the Shapiro-Wilks test. As each participant served as his own control, participants who were present at the recall visits were included in the analysis. A paired *t* test was used for the intragroup and between-group comparisons. The alpha error was 0.05.

### 2.10 Randomization, allocation concealment, and blinding

Randomization of half-mouth treatment and treatment order was generated by Microsoft Excel software. Allocation was concealed in opaque envelopes with serial numbers and was stored by a central registrar. The examiner, laboratory personnel, and statistician were blinded to the allocation. The allocation was concealed from the operator until the treatments.

## 3 Results

### 3.1 Experimental population and participant characteristics

**Figure 1:**
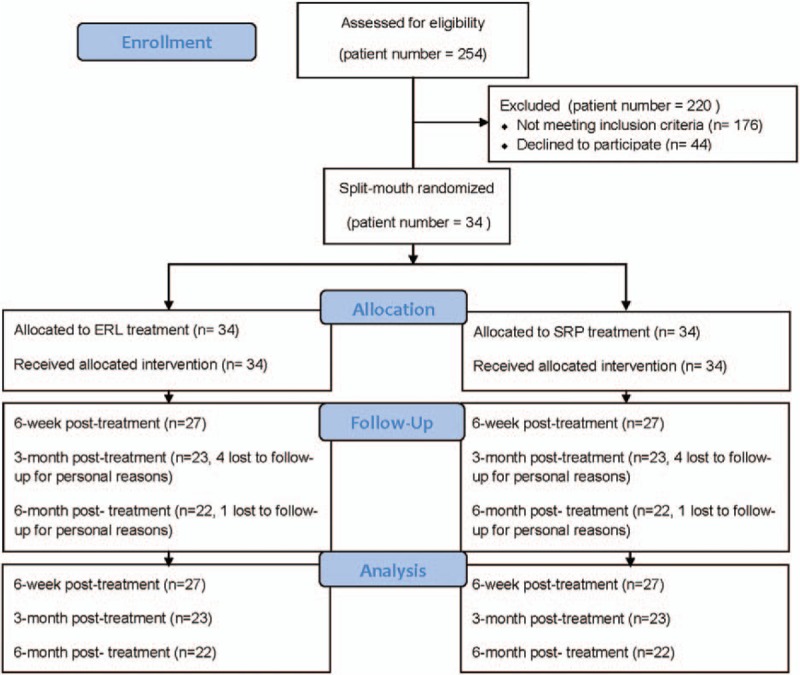
Flow chart presenting the outline of the study.

After 254 periodontal patients were screened, 34 participants received both treatments, and 27 of them attended the first recall visit. The mean age of the 27 participants was 43.6 ± 8.7 years (range 28–56 years), and 13 participants were male. Four participants were absent from the 3-month and 6-month recall visits, and 1 participant did not attend the 6-month recall visit. All absences were for personal reasons (Fig. 1). This study included 612 teeth and 2213 sites (Table 1).

**Table 1:**
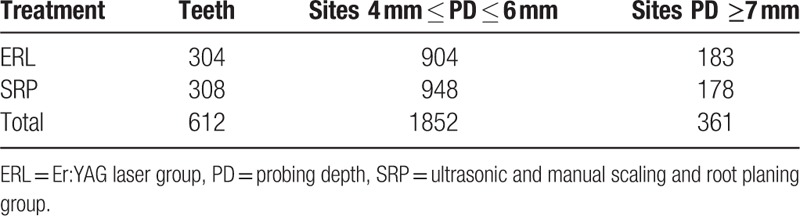
Tooth and site distributions divided according to treatment.

### 3.2 Clinical parameters

The healing process of both the Er:YAG and SRP groups was uneventful. No abscesses or acute infection symptoms were found during the observation period.

Table 2 shows the clinical parameters of sites with 4 mm ≤ PD ≤ 6 mm at baseline during the 6-month observation from 2 intervention groups. Both groups showed significant improvements in PD, BI, and CAL from baseline. Compared with Er:YAG treatment, SRP resulted in significantly greater reductions in PD at 6 weeks, 3 months, and 6 months post-treatment (*P* < .01, *P* = .04, and *P* = .01, respectively), in BI at 6 weeks post-treatment (*P* = .01), and in CAL at 6 weeks, 3 months, and 6 months post-treatment (*P* = .03, *P* = .04, and *P* < .01, respectively).

**Table 2:**
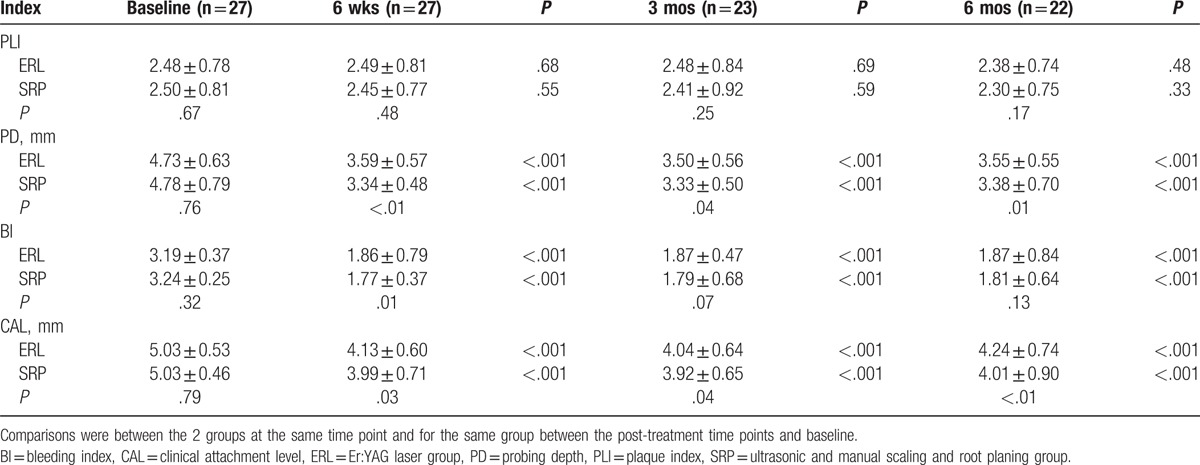
Clinical parameters for sites with 4 mm ≤ PD ≤ 6 mm from baseline to 6 months after treatment in the 2 groups.

Table 3 shows the clinical parameters of sites with PD ≥7 mm at baseline during the 6-month observation from the 2 intervention groups. Both groups showed significant reductions in PD, BI, and CAL from baseline. There were no significant differences in any clinical parameters between the 2 groups.

**Table 3:**
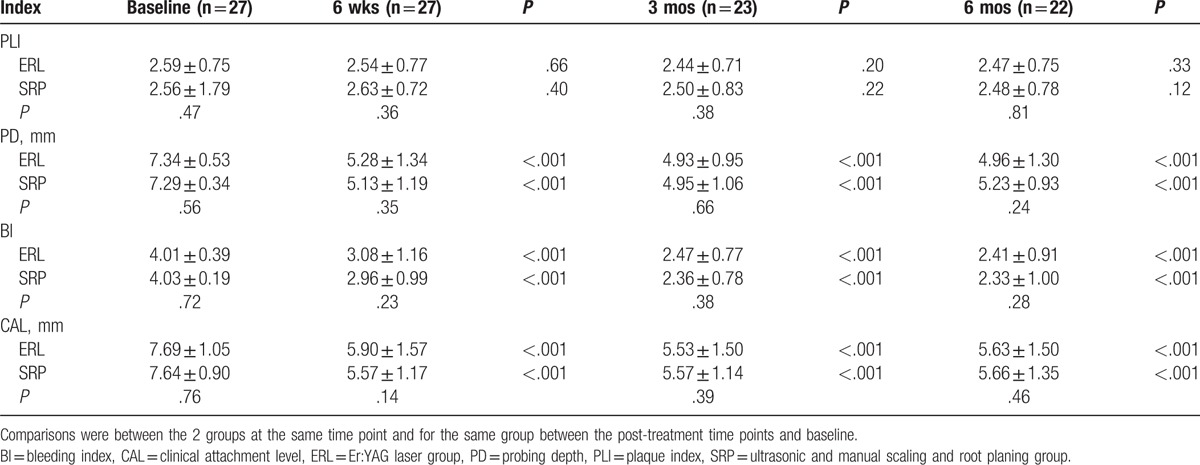
Clinical parameters for sites with PD ≥7 mm from baseline to 6 months after treatment in the 2 groups.

### 3.3 Microorganisms in GCF

**Figure 2:**
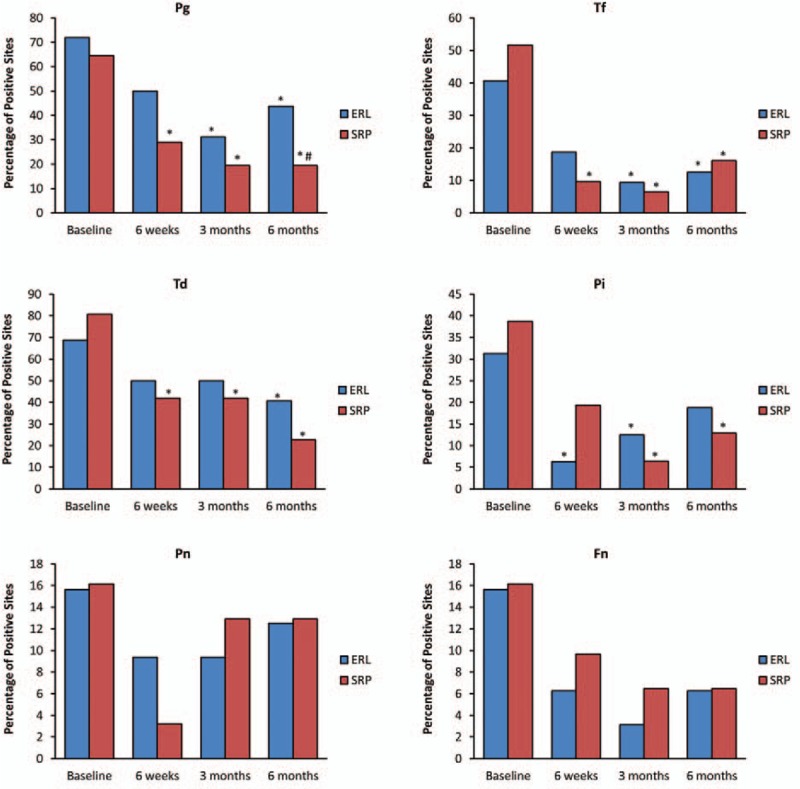
Percentage of sites positive in microbial testing for *Pg*, *Tf*, *Td*, *Pi*, *Pn*, and *Fn* from baseline to 6 months in the 2 groups. (∗) Indicates significance (*P* < .05) compared with baseline. (†) indicates significance (*P* < .05) between the 2 treatment groups. Er:YAG = Er:YAG laser group, SRP = combination of ultrasonic and manual scaling and root planing group.

A total of 692 samples were collected in this study, which included 348 in the laser group and 344 in the SRP group for 4 visits. *Pg*, *Tf*, *Td*, and *Pi* were significantly decreased after treatment, whereas *Pn* and *Fn* showed no significant differences before and after treatment. There was no significant difference between the 2 groups at any time point, except that the detection rate of *Pg* in the SRP group at 6 months post-treatment was lower than in the Er:YAG group (*P* = .04) (Fig. 2).

### 3.4 Calprotectin levels in GCF

**Figure 3:**
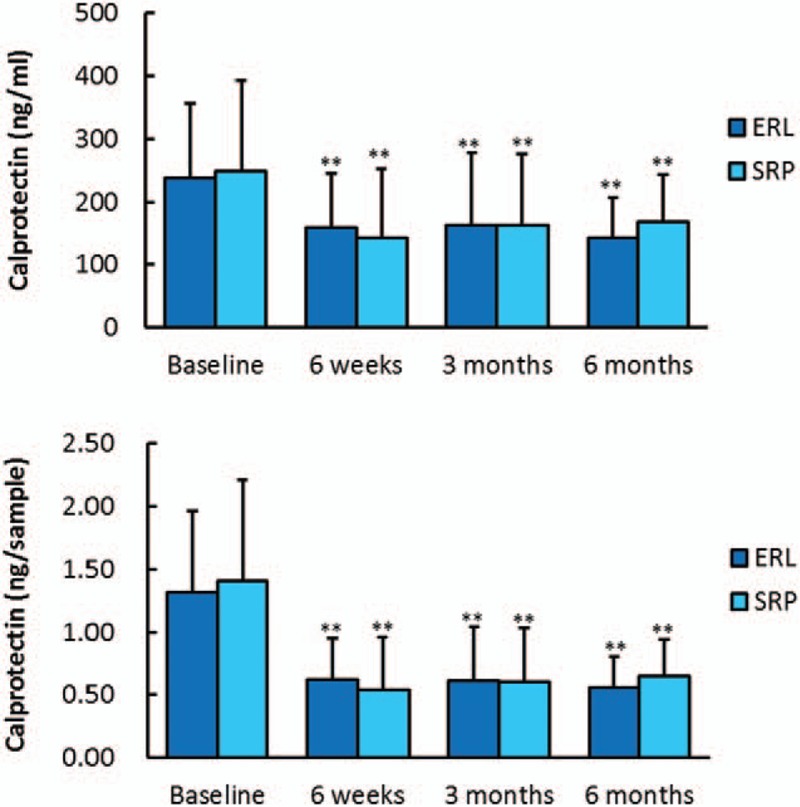
Concentration of calprotectin (ng/mL) and total amount of calprotectin (ng/sample) from baseline to 6 months in the 2 groups. (∗∗) Indicates significance (*P* < .01) compared with baseline. Er:YAG = Er:YAG laser group, SRP = combination of ultrasonic and manual scaling and root planing group.

For both groups, the concentration and total amount of calprotectin had decreased significantly at 6 weeks and at 3 and 6 months after treatment compared with baseline. At the same time points, there was no significant difference in the concentration or total amount of calprotectin between the Er:YAG and SRP groups (Fig. 3).

## 4 Discussion

In our study, both Er:YAG laser and conventional SRP treatments for chronic periodontitis achieved significant improvements in clinical parameters, microbiological effects, and calprotectin levels during the 6-month follow-up. For sites with 4 mm ≤ PD ≤ 6 mm at baseline, SRP resulted in a greater reduction in PD and CAL, which remained significant at 6 months post-treatment. For sites with PD ≥7 mm at baseline, the clinical parameters showed similar results between the 2 groups. The *Pg* detection rate was significantly lower in the SRP group at 6 months after treatment. There was no difference in calprotectin levels between the 2 groups at any time point.

Most of the previous studies were based on comparisons between Er:YAG laser monotherapy and either manual or ultrasonic SRP alone.^[11–13,15–17]^ In the present study, a combination of ultrasonic and hand instruments was used as a control group to simulate common clinical practice as closely as possible, and this combination could provide better results than either instrument alone.^[25]^ To better explore the application of Er:YAG laser, we grouped the sites with 4 mm ≤ PD ≤ 6 mm and PD ≥7 mm at baseline as mild pockets and deep pockets, respectively. For mild pockets, we found that SRP resulted in better improvement in PD and CAL compared with Er:YAG laser monotherapy, and the difference lasted until 6 months after treatment. One report by Soo et al,^[18]^ which also compared Er:YAG laser monotherapy with a combination of ultrasonic and hand instruments, showed that SRP resulted in significantly greater short-term improvement in clinical parameters. Interestingly, in that study, pockets with PD <4 mm were also included in the treatment and analysis, and the mean value of PD was even lower than our mild pockets. A study by Rotundo et al also compared Er:YAG laser monotherapy with the combination of ultrasonic and hand instruments, in which the mean value of PD in both groups was 5.2 mm, and less PD and CAL gain were also shown in the Er:YAG laser group. For deep pockets, our study showed that Er:YAG laser monotherapy resulted in comparable clinical improvement to SRP. Although we did not show more benefits for Er:YAG laser than SRP when applied to deep pockets, our results suggested that Er:YAG laser had more potential in deep pockets than in mild pockets. In a previous study comparing Er:YAG laser monotherapy and ultrasonic SRP, as the treated sites became deeper, the benefits of Er:YAG laser became more obvious,^[26]^ which indicated that without access limitations due to restrictions in physical size, Er:YAG laser treatment may have more potential in inaccessible areas. Some studies have tested new applications of Er:YAG laser in deep pockets, and the results were promising. One study reported that additional Er:YAG laser therapy for deep pockets alone (PD ≥4.5 mm) 1 week after ultrasonic SRP resulted in a smaller number and a lower percentage of bleeding on probing at a 12-month follow-up.^[27]^ Another recent study suggested that a combination of Er:YAG and neodymium-doped yttrium aluminium garnet laser treatment could achieve better improvement in clinical parameters for deep pockets (≥7 mm) than conventional SRP.^[28]^

In addition, we compared the detection rate of periodontal pathogens between the 2 treatments, and the results showed similar improvement for both treatments, except for a lower detection rate of *Pg* in the SRP group at 6 months post-treatment. Several studies have suggested that Er:YAG laser treatment has comparable bactericidal effectiveness to SRP.^[11,29,30]^ However, 1 in vitro study showed that Er:YAG laser may produce a rougher root surface than either hand or ultrasonic SRP.^[31]^ In our study, with the combination of ultrasonic and manual instruments, the root surfaces were even smoother than with either manual or ultrasonic debridement alone, which can prevent the recolonization of bacteria.^[32]^ That result might explain why our control group showed a lower *Pg* detection rate at 6 months post-treatment.

Moreover, we determined the levels of calprotectin in the GCF before and after the 2 treatments. To the best of our knowledge, this is the first study to compare calprotectin levels in GCF between Er:YAG laser and conventional SRP treatments. Calprotectin, which is also called myeloid-related protein or S100A8/A9, is a calcium-binding protein that is mainly produced by polymorphonuclear leukocytes, monocytes, macrophages, and epithelial cells, and is involved in the early immune response.^[33,34]^ It has been reported that a high calprotectin level correlates with deteriorating periodontal status and other biomarkers (interleukin [IL]-1β, prostaglandin E2, collagenase, and aspartate aminotransferase),^[35,36]^ and it can be reduced by successful periodontal treatments.^[37–40]^ Kaner et al^[37]^ observed that sites with a high level of calprotectin in GCF at 3 months post-treatment were more likely to show disease progression from 3 to 6 months post-treatment, indicating that the calprotectin level in GCF could be a useful biomarker for monitoring the effects of periodontal treatment at the site level. A recent study conducted by Eick et al^[41]^ evaluated the levels of several biomarkers, including calprotectin, IL-1β, matrix metalloprotease (MMP)-8, MMP-1, and tissue inhibitor of MMP-1, in the GCF of periodontitis patients before and after SRP. Furthermore, calprotectin in GCF at 3 and 6 months after SRP was the most differentiated biomarker between high and low-treatment response sites at 6 months after SRP, suggesting that calprotectin is a relatively sensitive biomarker to show the effects of periodontal therapy. In our study, the calprotectin level showed similar significant reductions after Er:YAG laser and SRP treatments, and the reduction remained for up to 6 months, indicating comparable effects of these 2 treatments in a more sensitive and predictive way.

There are limitations of the present study. First, only a limited number and type (systemically healthy nonsmokers with chronic periodontitis) of participants were enrolled. Second, microbiological effects were revealed by the detection rate of periodontal pathogens. Third, we detected only the level of calprotectin as a biomarker reflecting the host immune response. Finally, our study investigated only the most common application of the Er:YAG laser. In recent years, more advanced Er:YAG laser instruments have been developed, such as an Er:YAG laser instrument with an automatic calculus detection system,^[42]^ a fiberless Er:YAG laser system,^[43]^ and modified applications,^[27,28]^ and more studies are needed to evaluate their effects.

## 5 Conclusions

For mild pockets, common SRP may be still the preferred choice, whereas for deep pockets, Er:YAG laser treatment could be an effective alternative. More studies are needed to explore more advanced instruments and new application methods for Er:YAG laser therapy of periodontitis in deep pockets.

## Supplementary Material

Supplemental Digital Content

## References

[1] Sanz M van Winkelhoff AJ Periodontal infections: understanding the complexity: consensus of the Seventh European Workshop on Periodontology. J Clin Periodontol 2011;38 (suppl 11):3–6. 21323698 10.1111/j.1600-051X.2010.01681.x

[2] Lindhe J Westfelt E Nyman S Long-term effect of surgical/non-surgical treatment of periodontal disease. J Clin Periodontol 1984;11:448–58. 6378986 10.1111/j.1600-051x.1984.tb01344.x

[3] Arabaci T Cicek Y Canakci CF Sonic and ultrasonic scalers in periodontal treatment: a review. Int J Dent Hyg 2007;5:2–12. 17250573 10.1111/j.1601-5037.2007.00217.x

[4] Sherman PR Hutchens LJ Jewson LG The effectiveness of subgingival scaling and root planing. II. Clinical responses related to residual calculus. J Periodontol 1990;61:9–15. 2179516 10.1902/jop.1990.61.1.9

[5] Copulos TA Low SB Walker CB Comparative analysis between a modified ultrasonic tip and hand instruments on clinical parameters of periodontal disease. J Periodontol 1993;64:694–700. 8410606 10.1902/jop.1993.64.8.694

[6] Periodontology TAAO American Academy of Periodontology Statement on the efficacy of lasers in the non-surgical treatment of inflammatory periodontal disease. J Periodontol 2011;82:513–4. 21453136 10.1902/jop.2011.114001

[7] Aoki A Miura M Akiyama F In vitro evaluation of Er:YAG laser scaling of subgingival calculus in comparison with ultrasonic scaling. J Periodontal Res 2000;35:266–77. 11005154 10.1034/j.1600-0765.2000.035005266.x

[8] Folwaczny M Aggstaller H Mehl A Removal of bacterial endotoxin from root surface with Er:YAG laser. Am J Dent 2003;16:3–5. 12744404

[9] Akiyama F Aoki A Miura-Uchiyama M In vitro studies of the ablation mechanism of periodontopathic bacteria and decontamination effect on periodontally diseased root surfaces by erbium:yttrium-aluminum-garnet laser. Lasers Med Sci 2011;26:193–204. 20309597 10.1007/s10103-010-0763-3

[10] Feng XH Lu RF He L A short-term clinical evaluation of periodontal treatment with an Er:YAG laser for patients with chronic periodontitis: a split-mouth controlled study. Beijing Da Xue Xue Bao 2011;43:886–90. 22178840

[11] Lopes BM Theodoro LH Melo RF Clinical and microbiologic follow-up evaluations after non-surgical periodontal treatment with erbium:YAG laser and scaling and root planing. J Periodontol 2010;81:682–91. 20429647 10.1902/jop.2010.090300

[12] Badran Z Boutigny H Struillou X Clinical outcomes after nonsurgical periodontal therapy with an Er:YAG laser device: a randomized controlled pilot study. Photomed Laser Surg 2012;30:347–53. 22554048 10.1089/pho.2011.3215

[13] Malali E Kadir T Noyan U Er:YAG lasers versus ultrasonic and hand instruments in periodontal therapy: clinical parameters, intracrevicular micro-organism and leukocyte counts. Photomed Laser Surg 2012;30:543–50. 22824071 10.1089/pho.2011.3202

[14] Sculean A Schwarz F Berakdar M Periodontal treatment with an Er:YAG laser compared to ultrasonic instrumentation: a pilot study. J Periodontol 2004;75:966–73. 15341354 10.1902/jop.2004.75.7.966

[15] Lopes BM Marcantonio RA Thompson GM Short-term clinical and immunologic effects of scaling and root planing with Er:YAG laser in chronic periodontitis. J Periodontol 2008;79:1158–67. 18597597 10.1902/jop.2008.070600

[16] Sgolastra F Petrucci A Gatto R Efficacy of Er:YAG laser in the treatment of chronic periodontitis: systematic review and meta-analysis. Laser Med Sci 2012;27:661–73. 10.1007/s10103-011-0928-8 21553003

[17] Zhao Y Yin Y Tao L Er:YAG laser versus scaling and root planing as alternative or adjuvant for chronic periodontitis treatment: a systematic review. J Clin Periodontol 2014;41:1069–79. 25164559 10.1111/jcpe.12304

[18] Soo L Leichter JW Windle J A comparison of Er:YAG laser and mechanical debridement for the non-surgical treatment of chronic periodontitis: a randomized, prospective clinical study. J Clin Periodontol 2012;39:537–45. 22486380 10.1111/j.1600-051X.2012.01873.x

[19] Rotundo R Nieri M Cairo F Lack of adjunctive benefit of Er:YAG laser in non-surgical periodontal treatment: a randomized split-mouth clinical trial. J Clin Periodontol 2010;37:526–33. 20507376 10.1111/j.1600-051X.2010.01560.x

[20] Armitage GC Development of a classification system for periodontal diseases and conditions. Ann Periodontol 1999;4:1–6. 10863370 10.1902/annals.1999.4.1.1

[21] Turesky S Gilmore ND Glickman I Reduced plaque formation by the chloromethyl analogue of victamine C. J Periodontol 1970;41:41–3. 5264376 10.1902/jop.1970.41.41.41

[22] Mazza JE Newman MG Sims TN Clinical and antimicrobial effect of stannous fluoride on periodontitis. J Clin Periodontol 1981;8:203–12. 6947986 10.1111/j.1600-051x.1981.tb02031.x

[23] Baumgartner JC Siqueira JJ Xia T Geographical differences in bacteria detected in endodontic infections using polymerase chain reaction. J Endod 2004;30:141–4. 15055430 10.1097/00004770-200403000-00004

[24] Ashimoto A Chen C Bakker I Polymerase chain reaction detection of 8 putative periodontal pathogens in subgingival plaque of gingivitis and advanced periodontitis lesions. Oral Microbiol Immunol 1996;11:266–73. 9002880 10.1111/j.1399-302x.1996.tb00180.x

[25] Aspriello SD Piemontese M Levrini L Ultramorphology of the root surface subsequent to hand-ultrasonic simultaneous instrumentation during non-surgical periodontal treatments: an in vitro study. J Appl Oral Sci 2011;19:74–81. 21437474 10.1590/S1678-77572011000100015 PMC4245868

[26] Crespi R Capparè P Toscanelli I Effects of Er:YAG laser compared to ultrasonic scaler in periodontal treatment: a 2-year follow-up split-mouth clinical study. J Periodontol 2007;78:1195–200. 17608573 10.1902/jop.2007.060460

[27] Sanz-Sánchez I Ortiz-Vigón A Matos R Clinical efficacy of subgingival debridement with adjunctive Er:YAG laser in chronic periodontitis patients. a randomised clinical trial. J Periodontol 2015;86:527–35. 25543679 10.1902/jop.2014.140258

[28] Saglam M Koseoglu S Tasdemir I Combined application of Er:YAG and Nd:YAG lasers in treatment of chronic periodontitis. A split-mouth, single-blind, randomized controlled trial. J Periodontal Res 2017;52:853–62. 28332191 10.1111/jre.12454

[29] Derdilopoulou FV Nonhoff J Neumann K Microbiological findings after periodontal therapy using curettes, Er:YAG laser, sonic, and ultrasonic scalers. J Clin Periodontol 2007;34:588–98. 17555412 10.1111/j.1600-051X.2007.01093.x

[30] Milne TJ Coates DE Leichter JW Periodontopathogen levels following the use of an Er:YAG laser in the treatment of chronic periodontitis. Aust Dent J 2016;61:35–44. 10.1111/adj.12306 25630495

[31] Mishra MK Prakash S A comparative scanning electron microscopy study between hand instrument, ultrasonic scaling and erbium doped:Yttirum aluminum garnet laser on root surface: a morphological and thermal analysis. Contemp Clin Dent 2013;4:198–205. 24015009 10.4103/0976-237X.114881 PMC3757882

[32] Leknes KN Lie T Wikesjo UM Influence of tooth instrumentation roughness on subgingival microbial colonization. J Periodontol 1994;65:303–8. 8195973 10.1902/jop.1994.65.4.303

[33] Steinbakk M Naess-Andresen CF Lingaas E Antimicrobial actions of calcium binding leucocyte L1 protein, calprotectin. Lancet 1990;336:763–5. 1976144 10.1016/0140-6736(90)93237-j

[34] Ross KF Herzberg MC Calprotectin expression by gingival epithelial cells. Infect Immun 2001;69:3248–54. 11292747 10.1128/IAI.69.5.3248-3254.2001 PMC98283

[35] Kido J Nakamura T Kido R Calprotectin in gingival crevicular fluid correlates with clinical and biochemical markers of periodontal disease. J Clin Periodontol 1999;26:653–7. 10522776 10.1034/j.1600-051x.1999.261004.x

[36] Haririan H Andrukhov O Pablik E Comparative analysis of calcium-binding myeloid-related protein-8/14 in saliva and serum of patients with periodontitis and healthy individuals. J Periodontol 2016;87:184–92. 26447749 10.1902/jop.2015.150254

[37] Kaner D Bernimoulin JP Dietrich T Calprotectin levels in gingival crevicular fluid predict disease activity in patients treated for generalized aggressive periodontitis. J Periodontal Res 2011;46:417–26. 21488873 10.1111/j.1600-0765.2011.01355.x

[38] Kaner D Bernimoulin J Kleber B Gingival crevicular fluid levels of calprotectin and myeloperoxidase during therapy for generalized aggressive periodontitis. J Periodontal Res 2006;41:132–9. 16499716 10.1111/j.1600-0765.2005.00849.x

[39] Andersen E Dessaix IM Perneger T Myeloid-related protein (MRP8/14) expression in gingival crevice fluid in periodontal health and disease and after treatment. J Periodontal Res 2010;45:458–63. 20337885 10.1111/j.1600-0765.2009.01257.x

[40] Nakamura T Kido J Kido R The association of calprotectin level in gingival crevicular fluid with gingival index and the activities of collagenase and aspartate aminotransferase in adult periodontitis patients. J Periodontol 2000;71:361–7. 10776922 10.1902/jop.2000.71.3.361

[41] Eick S Mathey A Vollroth K Persistence of *Porphyromonas gingivalis* is a negative predictor in patients with moderate to severe periodontitis after nonsurgical periodontal therapy. Clin Oral Investig 2016;21:665–74. 10.1007/s00784-016-1933-x 27558382

[42] Badran Z Demoersman J Struillou X Laser-induced fluorescence for subgingival calculus detection: scientific rational and clinical application in periodontology. Photomed Laser Surg 2011;29:593–6. 21495861 10.1089/pho.2010.2951

[43] Yaneva B Firkova E Karaslavova E Bactericidal effects of using a fiber-less Er:YAG laser system for treatment of moderate chronic periodontitis: preliminary results. Quintessence Int 2014;45:489–97. 24701614 10.3290/j.qi.a31803

