# Supplementary material for: Comparison of clinical parameters, microbiological effects and calprotectin counts in gingival crevicular fluid between Er:YAG laser and conventional periodontal therapies: A split-mouth, single-blinded, randomized controlled trial
Source: Medicine (Baltimore). 2017 Dec 22;96(51):e9367. doi: 10.1097/MD.0000000000009367 (PMC5758231; doi:10.1097/MD.0000000000009367)
Supplement: Supplemental Digital Content [file medi-96-e9367-s001.doc]

Supplemental Digital Content. Table showing the sequences of the primers for six periodontal pathogens.

**A Comparison of the** **Clinical Parameters,** **Microbiological Effects and C**a**lprotectin Counts in Gingival Crevicular Fluid between Er:YAG Laser and Conventional Periodontal Therapy: A Split-mouth, Single-blinded, Randomized Controlled Trial**

Yue Wang, MD

| Supplemental Table. Species-specific and Universal Primers for PCR | | |
| --- | --- | --- |
| Species | Primer pair (5’-3’) | Base position  (amplicon length in bp) |
| Pg | AGG CAG CTT GCC ATA CTG CG  ACT GTT AGC AAC TAC CGA TGT | 404 |
| Tf | GCG TAT GTA ACC TGC CCG CA  TGC TTC AGT GTC AGT TAT ACC T | 641 |
| Td | TAA TAC CGA ATG TGC TCA TTT ACA T  TCA AAG AAG CAT TCC CTC TTC TTC TTA | 316 |
| Pi | TTT GTT GGG GAG TAA AGC GGG  TCA ACA TCT CTG TAT CCT GCG T | 575 |
| Pn | ATG AAA CAA AGG TTT TCC GGT AAG  CCC ACG TCT CTG TGG GCT GCG A | 804 |
| Fn | AGG GCA TCC TAG AAT TATG  GGG ACA CTG AAA CAT CTC TGT CTCA | 817 |
